# Advanced high resolution x-ray diagnostic for HEDP experiments

**DOI:** 10.1038/s41598-018-34717-9

**Published:** 2018-11-06

**Authors:** A. Y. Faenov, T. A. Pikuz, P. Mabey, B. Albertazzi, Th. Michel, G. Rigon, S. A. Pikuz, A. Buzmakov, S. Makarov, N. Ozaki, T. Matsuoka, K. Katagiri, K. Miyanishi, K. Takahashi, K. A. Tanaka, Y. Inubushi, T. Togashi, T. Yabuuchi, M. Yabashi, A. Casner, R. Kodama, M. Koenig

**Affiliations:** 1 Open and Transdisciplinary Research Initiatives, Osaka University, Suita, 565-0871 Japan; 2 Joint Institute for High Temperature RAS, Moscow, 125412 Russia; 3 LULI - CNRS, Ecole Polytechnique, CEA: Université Paris-Saclay; UPMC Univ Paris 06: Sorbonne Universités, F-91128 Palaiseau cedex, France; 4 FSRC “Crystallography and Photonics” RAS, Moscow, 119333 Russia; 5 Université de Bordeaux-CNRS-CEA, CELIA, UMR 5107, F-33405 Talence, France; 6 Graduate School of Engineering, Osaka University, Suita, Osaka 565-0871 Japan; 7 Institute of Laser Engineering, Osaka University, Suita, Osaka 565-0871 Japan; 8 Japan Synchrotron Radiation Research Institute, Sayo, Hyogo 679-5198 Japan; 9 RIKEN SPring-8 Center, Sayo, Hyogo 679-5148 Japan; 10 MEPhI, Moscow, 115409 Russia; 11 ELI-NP/IFIN-HH, Maqurele-Bucharest, 077125 Romania

## Abstract

High resolution X-ray imaging is crucial for many high energy density physics (HEDP) experiments. Recently developed techniques to improve resolution have, however, come at the cost of a decreased field of view. In this paper, an innovative experimental detector for X-ray imaging in the context of HEDP experiments with high spatial resolution, as well as a large field of view, is presented. The platform is based on coupling an X-ray backligther source with a Lithium Fluoride detector, characterized by its large dynamic range. A spatial resolution of 2 µm over a field of view greater than 2 mm^2^ is reported. The platform was benchmarked with both an X-ray free electron laser (XFEL) and an X-ray source produced by a short pulse laser. First, using a non-coherent short pulse laser-produced backlighter, reduced penumbra blurring, as a result of the large size of the X-ray source, is shown. Secondly, we demonstrate phase contrast imaging with a fully coherent monochromatic XFEL beam. Modeling of the absorption and phase contrast transmission of X-ray radiation passing through various targets is presented.

## Introduction

X-ray imaging is a fundamental diagnostic in the high energy density physics (HEDP) community, finding use in a wide range of fields including laboratory astrophysics^1,2^ and inertial confinement fusion research^3,4^. It enables the study of the temporal evolution of fast evolving phenomena such as shock compression of matter^5–7^ or plasma jets^8^, blast waves^9^, or hydrodynamic instabilities which are often opaque to visible light^10^. The recent development of very high-energy laser systems such as the NIF or LMJ^11^ opens new opportunities for experimental investigations of HEDP, necessitating both high spatial and temporal resolution X-ray imaging techniques to follow the microscale dynamics of these extreme states of matter. X-ray radiography has previously been used to image density distributions, instabilities^12^ and plasma shapes^13^. In all of these cases, the precision of the measurements is limited by the resolution of the platform. High temporal resolution is required for many processes and hence X-ray sources created by the interaction of a short-pulse laser with a metal target as well as X-ray Free Electron Lasers (XFELs) are often used. This has allowed processes evolving over several picoseconds or even femtoseconds to be studied^14,15^. On the other hand, measurements are currently limited by the spatial precision of X-ray detectors and methods^16^. The simplest and most often used schemes consist of a pinhole or point projection coupled with an image plate (IP) detector^17^ or X-ray CCD. One of the advantages of this technique is its large field of view, limited only by the size of image plate used, often on the order of centimeters or more. However, this setup can achieve, at best, a 15–25 µm spatial resolution^18^, which, for a great number of applications, is not sufficient to draw meaningful conclusions. For example, one of the biggest challenges, in HEDP at the current time, is turbulence, as it plays a vital role in star formation and the evolution of our galaxy. Ideally, the measure of the plasma density in this scenario should be performed from the spatial scale at which the laser energy is injected into the system (δ, the characteristic scale of the experiment) down to the Kolmogorov microscale *λ*_*k*_ where dissipation occurs^19^. Also of much interest, is the Rayleigh-Taylor instability (RTI), observed in astrophysics^20^, electro-hydrodynamics and notably in inertial confinement fusion (ICF) at the ablation front^21,22^. Theory and computational methods describe the development of the RTI with high accuracy for different materials and different initial conditions. Existing X-ray imaging methods, however, are not yet able to compete with theory because of their poor spatial resolution and therefore computational models cannot yet be validated or improved upon. Improved spatial resolution has been achieved recently, but always with the cost of a severely diminished field of view. Fresnel zone plates have been used to measure the spots of XFELs to a precision of 10 s of nm^23^, as well as the X-rays produced in laser target interactions^24^, yet their field of view is only several µm and hence are not compatible with HEDP experiments. Other methods, such as, Kirkpatrick-Baez systems are employed at XFELs^25^ or in ICF experiments^26^ to achieve a resolution of up to 8 µm, and a field of view on the order of tens of µm, yet for the latter case, this is still not sufficient to provide quality imaging for the capsule implosion. Talbot-Lau interferometers can give access to density gradients in a particular direction, but are still limited to a resolution of several tens of µm^27^ and a field of view of less than 1 mm. Schwarzschild microscopes have also been used for sub-micrometer imaging^28,29^ over a range of tens of µm, but are currently limited to lower photon energies in the EUV and soft X-ray range and therefore cannot be used to probe denser structures. Work has also been carried out on XFEL facilities to measure shock waves over scales of 100 s of µm with a resolution of 500 nm using phase contrast imaging^30^ and X-ray absorption edge imaging^31^. One may also improve the spatial resolution by decreasing the size of the pinhole, or to increase the magnification factor of the experimental platform. However, both of these methods drastically reduce the number of X-ray photons, worsen the signal-to-noise ratio and significantly lower the contrast of the radiography, often rendering detailed analysis impossible. Additionally, if the pinhole substrate is sufficiently heated, the material may expand, thus closing the pinhole and quenching the X-rays^32^. The resolution is therefore both strongly limited by the total number of available photons as well as the sensitivity of the detector used to measure them^8^. In this paper, we propose a new experimental scheme, capable of providing the number of photons, resolution and field of view, needed for a wide range of applications, adding to other X-ray imaging platforms currently in use.

The proposed platform makes use of Lithium Fluoride (LiF) crystal detectors. LiF is a well-known X-ray-sensitive material that is simple to use and can be made cheaply and in bulk^33^. Here, we use circular crystals with a diameter of 20 mm, however, much larger sizes are also commercially available, and hence there is no immediate limitation on the achievable field of view of this technique. The imaging properties are based on the photoluminescence of F-type color centers (CCs) which are generated under irradiation by photons with energy greater than 14 eV^34^. CCs are very stable at room temperature and may be read out any time after irradiation using a conventional fluorescent microscope^35^. In theory, the spatial resolution of an LiF detector corresponds to the size of the CCs, that is on the order of nanometres. However, in practice, for very hard X-ray radiation, photoelectron blurring is observed. This is because the energy of the incident photons is enough to generate secondary electrons, which in turn create CCs throughout the detector. For example, a spatial resolution of 1 µm has been reported for 10 keV radiation^36^. Moreover, since LiF is a passive detector and can be readily produced in any required quantity, its possible field of view is not limited as is the case for other X-ray detection techniques. LiF also has a large (at least 10^6^) dynamic range, is not sensitive to visible light and does not require electronic circuits. It has thus been used extensively in biological imaging^37^ and metrology^38^ with great success, and yet until now, has never been applied to X-ray diagnostics in the field of HEDP. In this paper, we present proof-of-principle results of a new experimental scheme including an LiF detector coupled to both an XFEL and a laser-produced X-ray source. A model of the transmission function of the monochromatic XFEL beam passing through modulated targets used for RTI experiments and subsequently incident on the LiF detector is also presented.

## Results

### Radiography with laser-produced X-ray source

**Figure 1:**
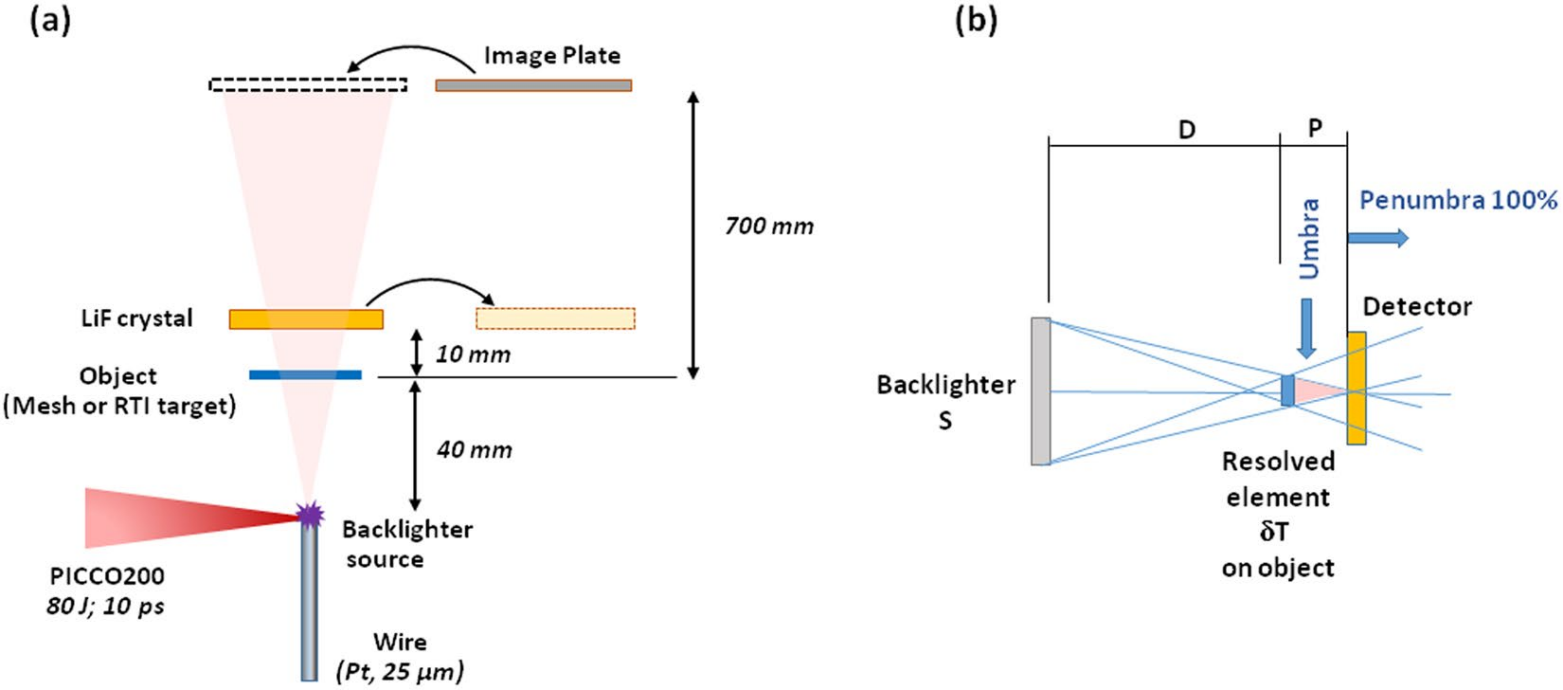
LULI2000 experimental setup: (**a**) The X-ray radiography scheme. (**b**) Schematic showing the geometrical limit on spatial resolution and the positioning of the detector.

**Figure 2:**
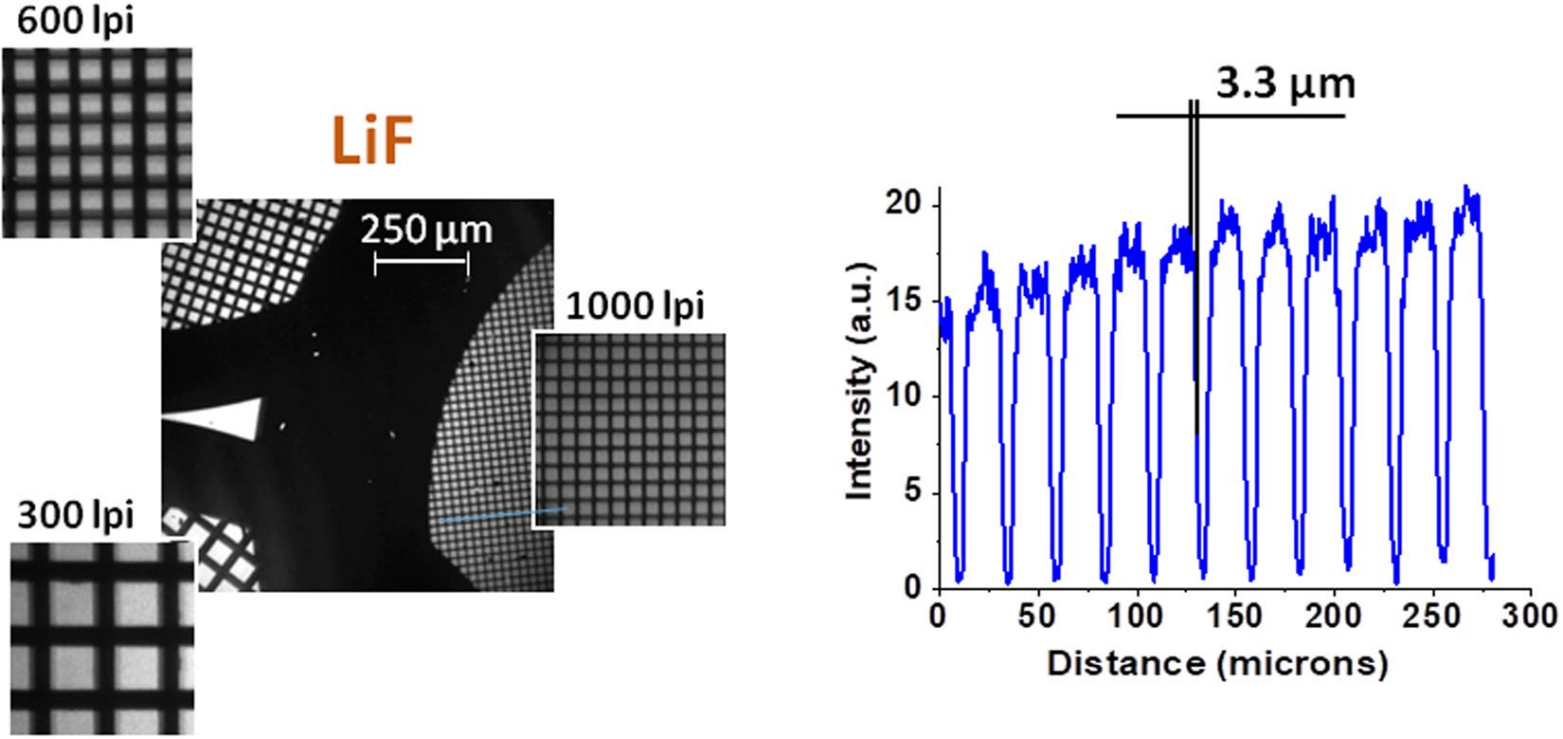
A radiography image of three Au meshes (300, 600 and 1000 lpi respectively), obtained with an LiF detector on one shot of the PICO2000 laser. An intensity profile taken from the lineout of the 1000 lpi mesh is also displayed.

The experiment was carried out at the LULI2000 facility using the setup shown in Fig. 1(a). Detailed descriptions of the laser beams, diagnostics and sample setup are given in the methods section. In Fig. 1(b), the formation of a shadow image of an opaque element, with a size much smaller than the size of the backlighter, is shown schematically. The maximum spatial resolution theoretically possible, δx, is defined by the distance over which the umbra (full shadow) disappears and the penumbra (blurring) becomes 100%. It is linked to the geometry of the setup by the relation: P = δx·D/S, where S is the size of the backlighter source and distances P and D are defined in Fig. 1(b). Assuming that the source has the same size as the wire itself, S = 25 µm, then δx = 5 µm. In Fig. 2(a), the image of the 300 lpi, 600 lpi and 1000 lpi meshes obtained on the LiF detector in one shot of the PICO2000 laser beam, is shown. As LiF is not sensitive to visible light, no filtering was required; therefore, all radiation from the backlighter above the threshold energy of 14 eV was recorded by the detector. All three meshes are well resolved and the whole image exhibits a qualitatively good contrast. Traces of intensity taken through the crop of the 1000 lpi mesh image demonstrate a spatial resolution of 3.3 µm (Fig. 2(b)). This value is in agreement with our prediction based on a very simple geometric calculation.

**Figure 3:**
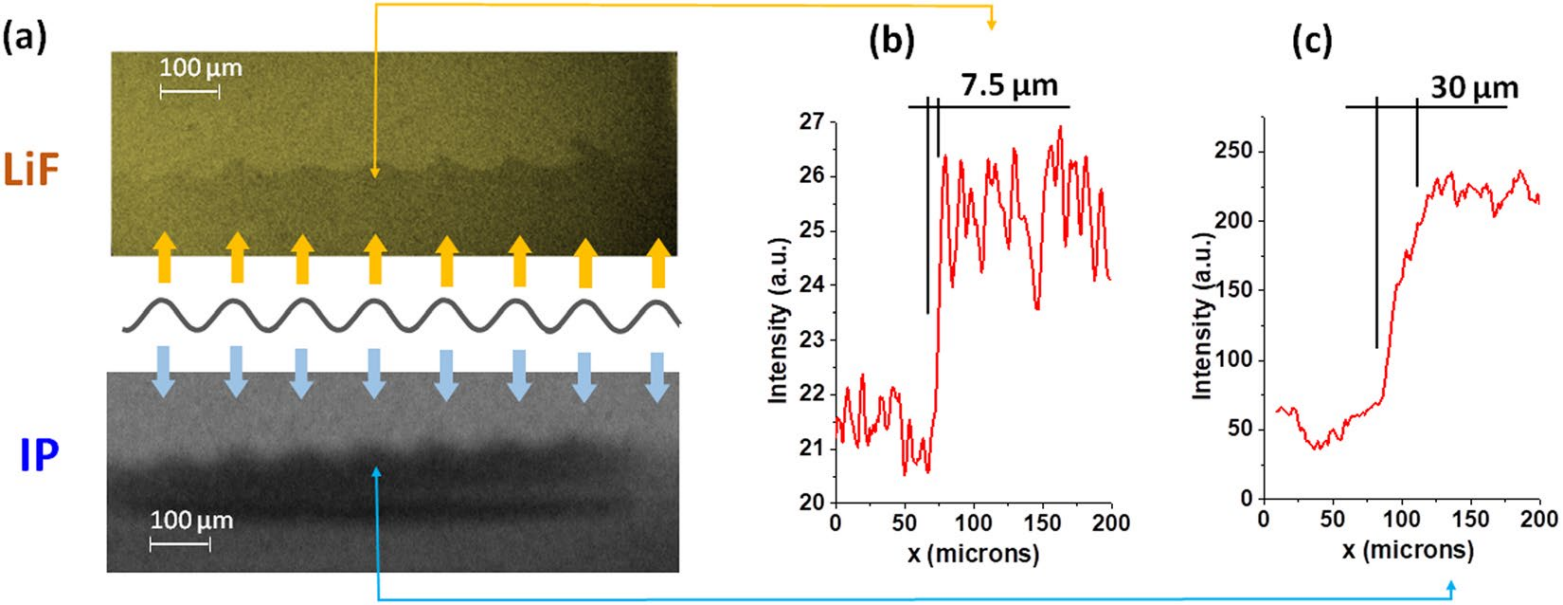
(**a**) A comparison of the transmission images of the RTI target, obtained with both LiF and IP detectors (thick arrows show relevant peaks of ripple surface). Panels (b) and (c) indicate intensity the profile along thin arrows in the LiF image and IP image respectively.

The same setup was then used to perform radiography on a real target, designed for use in a typical RTI experiment. A complete description of these targets may be found in the methods section. We compared the transmission image of an RTI target obtained on the LiF and on a common detector used in this type of experiment, an image plate (IP) (see Fig. 3(a). In order to obtain the IP image, the LiF detector is removed and the IP is placed at a distance of 700 mm from the target (see Fig. 1(a)). Traces of intensity profiles taken along the white lines clearly show that the interface is resolved significantly better with the LiF (trace in Fig. 3(b)) compared to with the IP (Fig. 3(c)): 7.5 µm and 30 µm respectively. It must be noted however, that the IP does show a higher contrast image because of the difference in response of the two detectors in the 4–5 keV spectral range. This indicates that although the spatial resolution is five times higher with the LiF detector, a comparatively higher X-ray flux is nevertheless required. The quality of the results displayed here, however, show that the laser energy provided by the LULI facility does indeed meet these requirements. We estimate that, using this configuration there are on the order of 5·10^7^ photons/mm^2^ according to previous papers^15,39^ incident on the LiF. Our preliminary measurements with synchrotron sources show that an LiF colorization threshold is of ~(1–5)·10^7^ photons/mm^2^ for photon energy range around 5 keV. Therefore in our experiments the X-ray flux had to be at an upper bound on the minimum flux needed for this detector. Since X-ray photon numbers scale with laser energy, facilities such as LMJ PETAL or NIF ARC, which have over 10 times more available laser energy, as well as XFELs, with a single shot flux in excess of 10^11^ photons/mm^2^, therefore comfortably meet these flux requirements. Additionally, in the experiment described here, the source-to-detector distance was 50 mm because of experimental constraints, but could readily be lowered in cases of low photon flux. A more detailed study on the response of LiF would be required to assess the feasibility of using this detector at smaller facilities, but is beyond the scope of this work.

### X-ray imaging with monochromatic fully coherent XFEL source

In addition to the large increase in brightness of XFEL radiation, the beam is also coherent and well collimated, thus allowing the possibility to perform phase contrast imaging (PCI) rather than simple absorption radiography. This technique is based on the propagation of phase-shifted X-ray photons induced by a density gradient; spatially coherent radiation is deflected from regions of higher density to regions of lower density. Due to the lack of coherence, and relatively modest numbers of divergent photons, laser-produced X-ray sources are not well suited to PCI. XFEL and synchrotron radiation, however, does not have these drawbacks.

**Figure 4:**
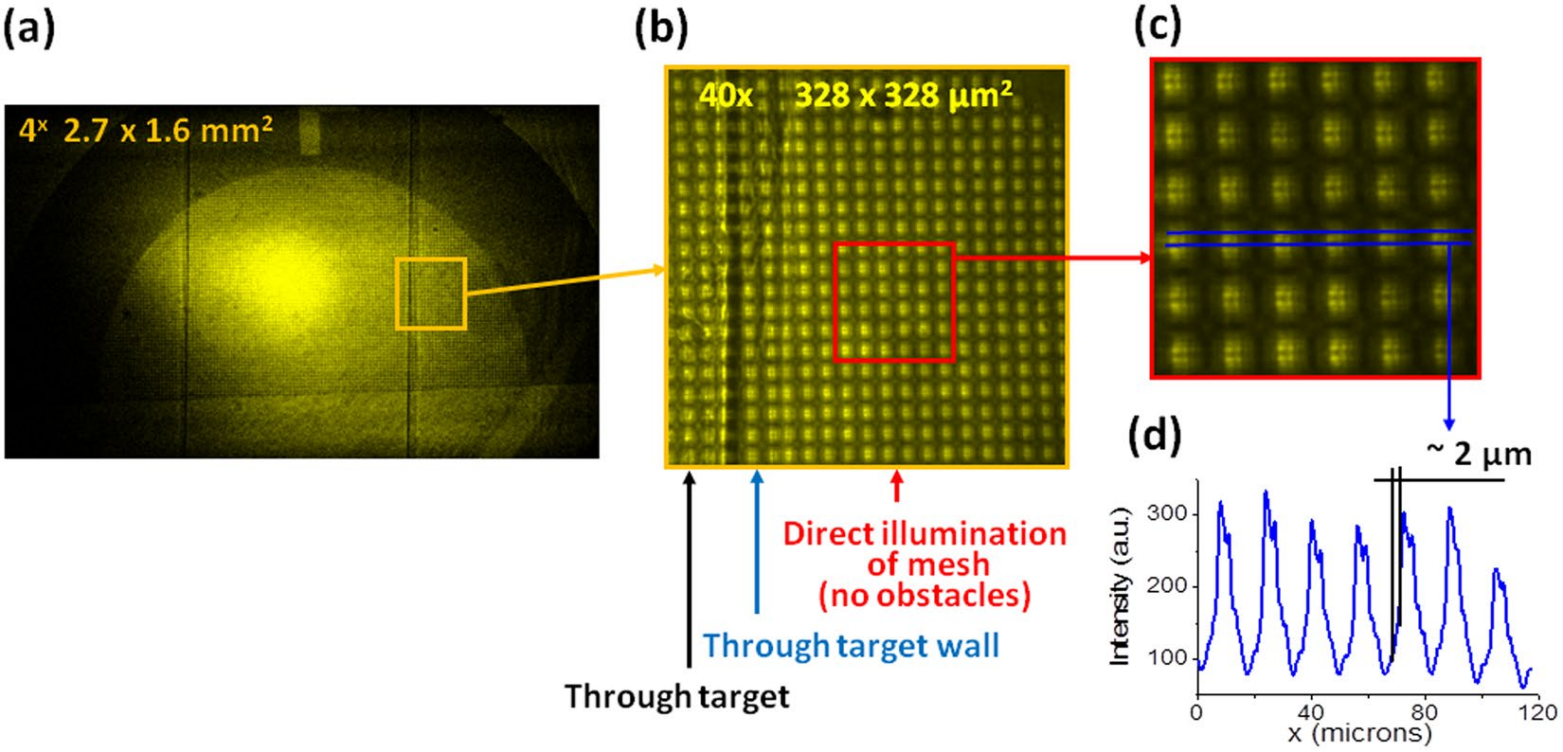
The image of 1500 lpi mesh obtained on LiF for the evaluation of an instrumental spatial resolution: Panel (a) contains a large field of view photoluminescent image, observed with a 4X microscope objective. Panel (b) shows part of the full image with different elements of target, observed with objective 40X, while panel (c) shows an enlarged crop of this image, in which the diffraction pattern in open areas of the mesh is clearly seen. Panel (d) contains an intensity profile taken from the cropped image.

To that end, an experiment at the SACLA XFEL was performed in order to characterize the new experimental scheme using a higher photon count and employing PCI rather than simple radiography. The experiment was carried out at beam line BL3, experimental hutch EH5 and a full description of the X-ray beam parameters used can be found in the methods section. Initially a 1500 lpi mesh was attached to the front surface of the RTI target and the LiF detector placed at a distance of 120 mm from the target chamber center. The target was illuminated with one shot of the XFEL beam at full energy, with the resulting LiF images shown in Fig. 4. Panel (a) contains a large field of view photoluminescent image, observed with a 4X microscope objective. Panel (b) represents part of a full image with different elements of the RTI target measured, this time with a 40X objective. Different elements of the RTI target are easily distinguished even through the mesh screening. The effect of the phase contrast enhancement allows the wall of the shock tube to be seen clearly. Panel (c) is an enlarged crop of image (b), which shows that in open areas of the mesh, the diffraction pattern is resolved. The intensity profile, presented in panel (d) shows that a spatial resolution of at least 2 µm was obtained (the spatial resolution measurement was limited by the diffraction fringes within the grid).

**Figure 5:**
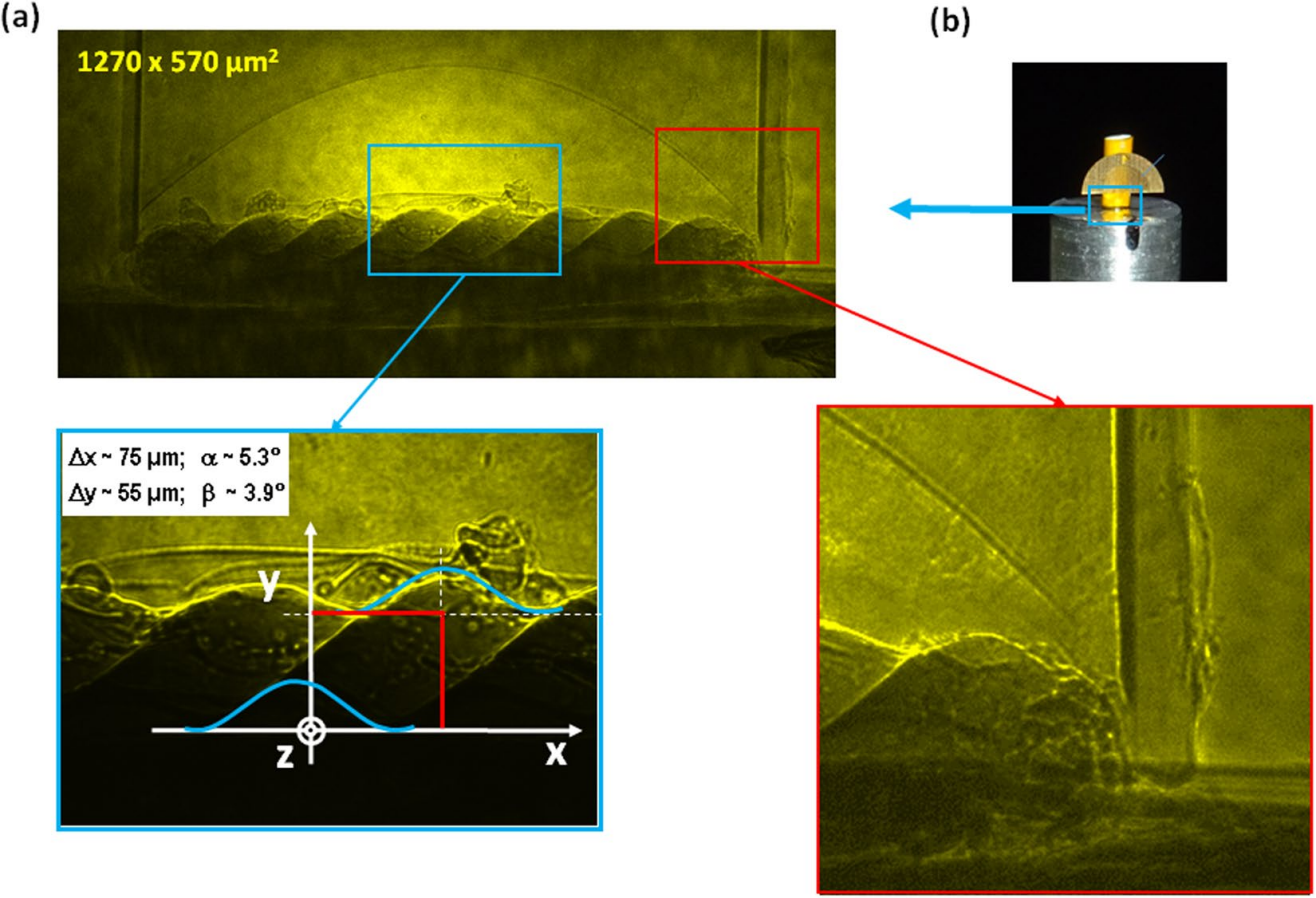
(**a**) Photoluminescence image of the RTI target interface obtained on a single shot of the SACLA XFEL using an LiF detector. The effect of the phase contrast enhancement is clearly seen. Two parts of the image are enlarged to better see the structural details of the target and multiple small inhomogeneities. The left hand panel shows schematically a measurement of the angular misalignment of the RTI target. Z is the axis of the XFEL beam, α and β are the angles of rotation around horizontal X and vertical Y axis, respectively. Panel (b) shows a view of the target indicating the part which is irradiated by the XFEL.

Next, the radiography was performed on the real RTI target using the same conditions, with the results being shown in Fig. 5. For clarification, panel (b) shows exactly which part of the RTI target was irradiated with the XFEL beam. The effect of the phase contrast imaging is clearly seen and the spatial resolution allows the observation of all the structural details of the target as well as multiple small inhomogeneities. The images show the presence of an air bubble attached to the rippled surface. The boundary between the air cupola and plastic foam is visible despite of low absorption of 10 keV radiation in the foam. A visible inclination of the ripples on the interface in Fig. 5(a) indicates the existence of a measurable angular misalignment of the target relative to the axis of the XFEL beam. This is shown schematically in the Fig. 5. Here, the experiment misalignment is shown to be of the order of a few degrees. Radiography using an LiF detector can therefore provide information on the angle of the target that can be taken into account in data processing. These images show the potential of the LiF detector platform compared to previous techniques employed within the field. The improved resolution using an XFEL rather than a laser-produced X-ray source is of course expected, however, and should not be seen as unique to the LiF detector. Rather, these results illustrate the full potential of this type of detector under optimal conditions.

**Figure 6:**
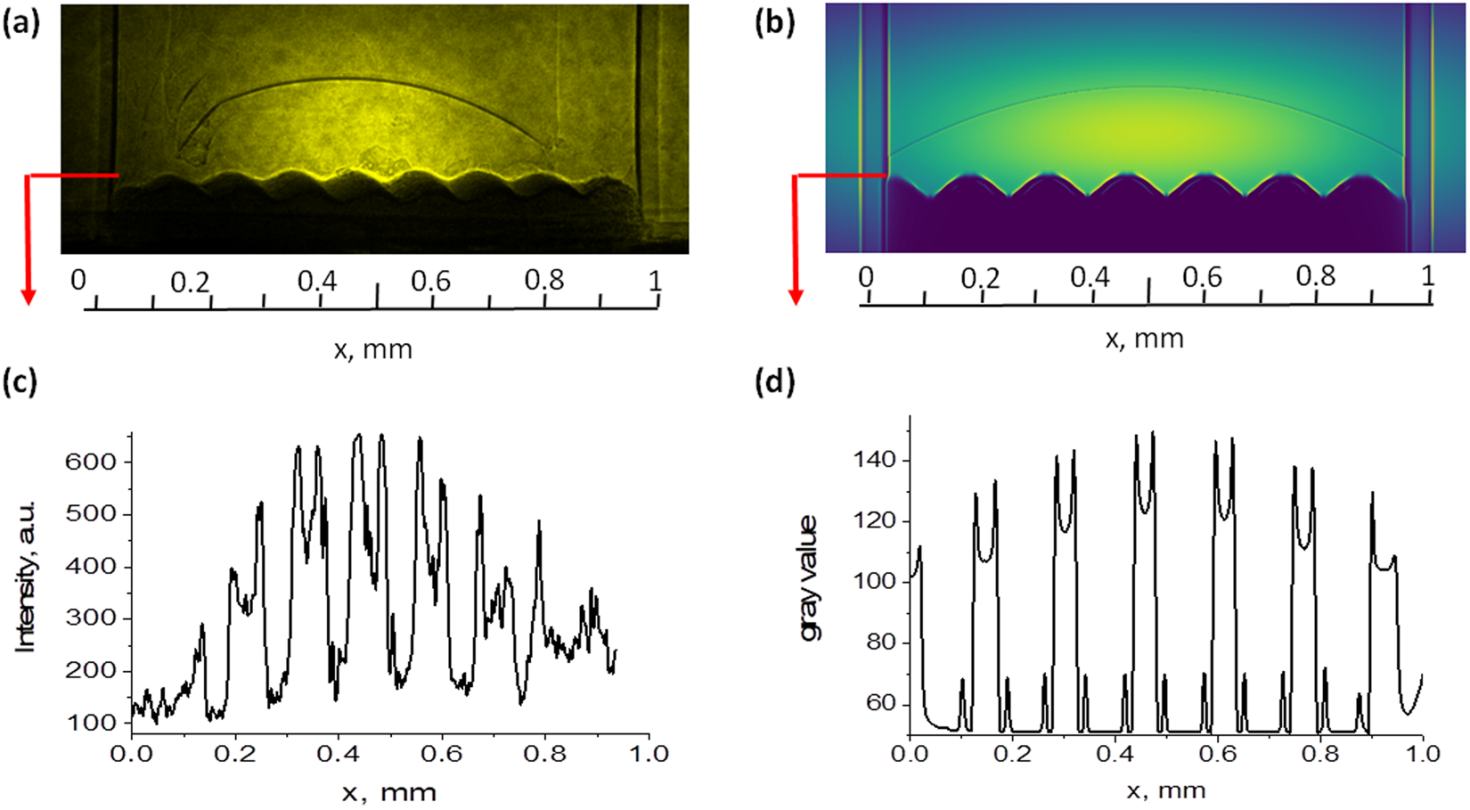
Comparison of experimental image obtained on the LiF crystal with modelling with lineout taken across rippled target edge. Phase contrast enhancement is observed in both cases.

The experimental results are also compared to a model of the idealized case of the radiography of the RTI target using a hard X-ray backlighter (monochromatic – spectral bandwidth is δ-function, fully coherent) with the results shown also in Fig. 6. The details of the model employed can be found in the methods section. One of its primary capabilities is the ability to determine whether PCI is expected for a given set of X-ray source and geometrical parameters. PCI is indeed predicted by the model for the experiment described here, although one notes a higher phase contrast enhancement than that measured experimentally on the LiF detector. This is inevitable however, due to the idealized approximations included for both the X-ray beam and the target. Aside from this, the model recreates the experimental image very well with the relative absorptions of the different sections of the target, well taken into account. Planning future X-ray imaging experiments will therefore be aided greatly by this tool. Not only will one have the ability to predict whether PCI will be possible with the given probe beam parameters, the contrast of the radiographic image may also be well predicted and therefore optimized by considering the design of the target.

## Discussion

The results demonstrate that the LiF detector, coupled with an X-ray backlighter source, represents an improvement on the experimental X-ray radiography platform, boasting a sufficiently large field of view (several mm) to image an entire HEDP experiment, with a high spatial resolution. First applied for imaging with non-coherent quasi-monochromatic laser-produced plasma X-rays, the platform reduces the amount of penumbra blurring. Experiments on imaging test-meshes and RTI targets in static conditions show that a spatial resolution of ~3.3 µm can be obtained with a backlighter size of ~25 µm. A second experiment employing phase contrast imaging with a coherent monochromatic XFEL beam significantly increases the visibility of inhomogeneities in low absorption objects. Coupling with the LiF detector allows the imaging of objects with spatial resolution better than 2 µm across the full aperture of the XFEL beam. These results have important consequences for future experiments across a wide range of areas in HEDP. LiF detectors could used on ICF experiments at large laser facilities, such as the NIF or LMJ-PETAL, in order to vastly increase the resolution available for imaging the small-scale structures and instabilities with the imploding fusion core, thus paving the way to better understand the dynamics of the system and enabling the validation of various models. Additionally, experiments investigating RTI instabilities in order to understand crucial astrophysical phenomena, such as supernovae remnants, would benefit greatly from the higher resolution and field of view enabled by this experimental platform. Moreover, the combination of spatial and temporal resolution, could be used for a range of applications in the field of shock physics. With a resolution smaller than typical grain sizes in most polycrystalline samples, LiF detectors open up the possibility of observing the effect of grain boundaries and orientations on shock propagation through samples. In general, coupled with their low cost, and ease of use, LiF detectors represent a vast improvement on the image plate detectors that are in widespread use in the field today.

## Methods

### RTI Targets

**Figure 7:**
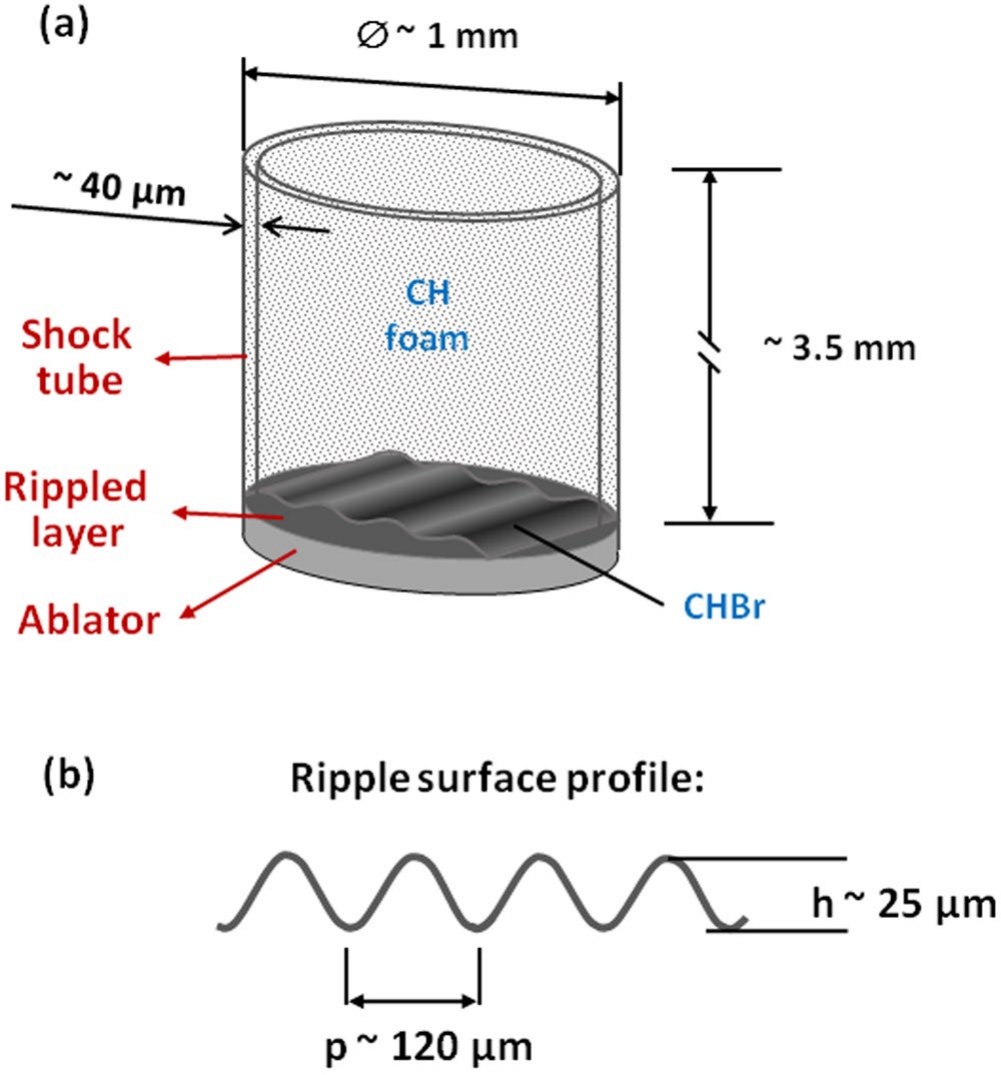
(**a**) A sketch of the RTI target, used to test the radiography platform. The material composition and the dimensions of the targets are shown. (**b**) The parameters of the pre-imposed ripples on the surface.

The targets used in this work consist of a layer of brominated plastic, attached to a thin (~1 µm) gold foil used as a radiation shield, a plastic ablator and a thin cylindrical shock tube, filled with low density plastic foam. Ripples with sinusoidal profile are pre-imposed on the entire surface of the brominated plastic layer. A sketch of the target is shown in Fig. 7(a). The shock tube is made from polyamide, with a diameter of ~1 mm, a height is of ~3.5 mm, and a wall thickness of ~40 µm. The initial profile of the ripples on the brominated layer have a wavelength of ~120 µm, with a peak-of-valley depth of ~20 µm (Fig. 3(a)). The density of the brominated layer (elemental composition C8H7Br) is 1.54 g/cc (3% atomic fraction), while the density of the CH foam is of 200 mg/cc. The bromine, buried into the profiled layer, increases the absorption and enhances the contrast of the radiography.

### Radiography with laser-produced X-ray source

The experiment was carried out at the LULI2000 facility. The X-ray backlighter source was created by the interaction of the PICO2000 laser pulse (80 J, 10 ps) with the tip of a platinum wire of 25 µm-diameter. Estimated X-ray yield for these laser parameters in the spectral range of 4.5–4.8 keV (K_α_ - 4.51 keV and He_α_ - 4.75 keV) is of the order of 10^11^ photons in a 4π solid angle. The backlighter target was placed at a distance, D = 40 mm, from the object to be radiographed. Three gold meshes with 300, 600 and 1000 lines per inch (lpi) were used to measure the resolution of the platform as well as real targets commonly used in RTI experiments. The LiF detector (diameter 20 mm and thickness 2 mm) was placed at a distance, P = 10 mm, from the object.

### X-ray imaging with monochromatic fully coherent XFEL source

The experiment was carried out at SACLA at beam line BL3, experimental hutch EH5. The XFEL beam had a photon energy of 10 keV, a pulse energy at the exit of the undulator of ~490 μJ/pulse, a divergence of 2.4 µrad, and a FWHM spot size on the target of ~1 mm. The LiF detectors used had a diameter of 20 mm and a thickness of 2 mm. Photoluminescence images recorded on the LiF were observed by scanning a confocal fluorescent Nikon C2 microscope with a laser excitation wavelength of 445 nm and magnifications of 4X, 10X and 40X.

### Modeling the propagation of XFEL radiation through RTI targets

For our modeling, we consider a beam with the same initial parameters described in the previous section. The modeling of the absorption and phase contrast images was provided using the software framework for coherent and partially coherent X-ray wavefront propagation simulations, WavePropaGator (WPG)^40^. This software package is a development of previous calculation methods and is the most relevant for FEL applications as well as being open access^41^.

For small emission and observation angles, the propagation of the transverse components of the electric field, *E*_⊥_, in free space, from a point, ***r***_**1**_, to a second point, ***r***_**2**_, can be described in terms of the Huygens−Fresnel principle, in the frequency domain, as an integral by integration over the plane ***Σ***_**1**_, perpendicular to the *z*-axis (beam axis).

1 $${E}_{\perp }({r}_{2},\,\omega \,)\approx -\frac{i\omega }{2\pi c}{\iint }_{{{\rm{\Sigma }}}_{1}}{E}_{\perp }({r}_{1},\omega )\frac{{e}^{-\frac{i\omega |{r}_{2}-{r}_{1}|}{c}}}{|{r}_{2}-{r}_{1}|}d{{\rm{\Sigma }}}_{1}.$$

The wavefield at the observation plane can be written in a more general form as

2 $${E}_{\perp }({x}_{2},{y}_{2},\omega )\approx \iint d{x}_{1}d{y}_{1}{\boldsymbol{K}}({x}_{2},{y}_{2},{x}_{1},{y}_{1},\omega ){E}_{\perp }({x}_{1},{y}_{1},\omega ),$$

where the kernel ***K*** (*x*_2_, *x*_1_, *y*_2_, *y*_1_, *ω*) is given as

3 $${\boldsymbol{K}}({x}_{2},{y}_{2},{x}_{1},{y}_{1},\omega )\approx -\frac{ik}{2\pi z}{e}^{\frac{ik}{2z}[{({x}_{2}-{x}_{1})}^{2}+{({y}_{2}-{y}_{1})}^{2}]}.$$

Propagation through most X-ray optical elements can be presented in the form of the convolution integral as in equation (2). Using the Fourier optics modular approach, a complete beamline can be described as a set of propagators corresponding to individual optical components, which can be then numerically solved by means of a 2D FFT algorithm. The representation in equation (3) also enables the analytical treatment of the quadratic phase term, allowing for the free-space propagation simulations with a reduced sampling rate. This dramatically improves the technical feasibility and robustness of the Fourier optics method.

Many of the optical components of an X-ray beamline can be described as thin optical elements, i.e. as linear filters that change the wavefield amplitude and/or phase in the plane normal to the propagation direction. For thin optical elements the kernel (3) can be represented as

4 $${\boldsymbol{K}}({x}_{2},{y}_{2},{x}_{1},{y}_{1},\omega )={\boldsymbol{T}}({x}_{1},{y}_{1},\omega )\delta ({x}_{1}-{x}_{2})\delta ({y}_{1}-{y}_{2}),$$

where ***T*** (*x*_1_, *y*_1_, *ω*) is a complex transmission function. For example, the transmission function for a modeling cylinder (with radius R, and center at *x*_0_) is given by

5 $${\boldsymbol{T}}(x,y,\omega )=\{\begin{array}{c}{e}^{-ik[2\ast \sqrt{{R}^{2}-{(R-(x-{x}_{0}))}^{2}}]},if\,|x-{x}_{0}|\le R\\ 1,if\,|x-{x}_{0}| > R\end{array}.$$

For modeling complex objects, one can build a 3D numerical model of the object and integrate it to obtain the 2D transmission function:

6 $${\boldsymbol{T}}(x,y,\omega )=\int {\boldsymbol{T}}(x,y,z,\omega )dz.$$

where, the z – axis is perpendicular to the observation plane.

Using this formula and the optical constants calculated with the xraylib software library^19^ we can build the transmission plane of our modeling object. The RTI target was considered as consisting of four different elements: a cylinder with thin walls, foam, an air cupola and a disk with a rippled surface. For each element, a 3D model and a complex 2D transmission plane, including absorption and phase shift, was built. The SACLA XFEL beam was modeled as a Gaussian beam object in WPG. After building the incident beam and the transmission plane, we propagate the wavefront using a free space propagator over a distance of 120 mm to the observation plane. This procedure was repeated for each element of the target.

## References

[1] Chabrier G, Douchin F, Potekhin AY (2002). Dense astrophysical plasmas. J. Phys. Condens. Matter.

[2] Remington BA, Drake RP, Ryutov DD (2006). Experimental astrophysics with high power lasers and Z pinches. Rev. Mod. Phys..

[3] Kodama R (2001). Fast heating of ultrahigh-density plasma as a step towards laser fusion ignition. Nature.

[4] Lindl JD (2004). The physics basis for ignition using indirect-drive targets on the National Ignition Facility. Phys. Plasmas.

[5] Ravasio A (2008). Hard x-ray radiography for density measurement in shock compressed matter. Phys. Plasmas.

[6] Brambrink E (2009). X-ray source studies for radiography of dense matter. Phys. Plasmas.

[7] Antonelli L (2017). Laser-driven shock waves studied by x-ray radiography. Phys. Rev. E.

[8] Diziere A (2015). Formation and propagation of laser-driven plasma jets in an ambient medium studied with X-ray radiography and optical diagnostics. Phys. Plasmas.

[9] Kuranz CC (2010). Spike morphology in blast-wave-driven instability experiments. Phys. Plasmas.

[10] Nagel SR (2017). A platform for studying the Rayleigh–Taylor and Richtmyer–Meshkov instabilities in a planar geometry at high energy density at the National Ignition Facility. Physics of Plasmas.

[11] Casner A., Caillaud T., Darbon S., Duval A., Thfouin I., Jadaud J.P., LeBreton J.P., Reverdin C., Rosse B., Rosch R., Blanchot N., Villette B., Wrobel R., Miquel J.L. (2015). LMJ/PETAL laser facility: Overview and opportunities for laboratory astrophysics. High Energy Density Physics.

[12] Smalyuk VA (2017). Hydrodynamic instability growth of three-dimensional modulations in radiation-driven implosions with “low-foot” and “high-foot” drives at the National Ignition Facility. Phys. Plasmas.

[13] Schelkovenko TA (2001). Radiographic and spectroscopic studies of X-pinch plasma implosion dynamics and x-ray burst emission characteristics. Phys. Plasmas.

[14] Brambrink E (2009). Direct density measurement of shock-compressed iron using hard x rays generated by a short laser pulse. Phys. Rev. E.

[15] Park H-S (2009). High-energy Kα radiography using high-intensity, short-pulse lasers. Phys. Plasmas.

[16] Kuranz CC (2009). Two-dimensional blast-wave-driven rayleigh–taylor instability: experiment and Simulation. The Astrophysical Journal.

[17] Gales SG, Bentley CD (2004). Image plates as x-ray detectors in plasma physics experiments. Rev. Sci. Instrum..

[18] Brambrink E (2016). Short-pulse laser-driven x-ray radiography. High Power Laser Science and Engineering.

[19] Zhou Y (2007). Unification and extension of the similarity scaling criteria and mixing transition for studying astrophysics using high energy density laboratory experiments or numerical simulations. Phys. Plasmas.

[20] Cabot WH, Cook AW (2006). Reynolds number effects on Rayleigh–Taylor instability with possible implications for type Ia supernovae. Nat. Phys..

[21] Martinez DA (2015). Evidence for a Bubble-Competition Regime in Indirectly Driven Ablative Rayleigh-Taylor Instability Experiments on the NIF. Phys. Rev. Lett..

[22] Casner A (2015). Probing the deep nonlinear stage of the ablative Rayleigh-Taylor instability in indirect drive experiments on the National Ignition Facility. Phys. Plasmas.

[23] David C (2011). Nanofocusing of hard X-ray free electron laser pulses using diamond based Fresnel zone plates. Sci. Rep..

[24] Do A (2017). High-resolution quasi-monochromatic X-ray imaging using a Fresnel phase zone plate and a multilayer mirror. Rev. Sci. Instrum..

[25] Yumoto H (2013). Focusing of X-ray free-electron laser pulses with reflective optics. Nat. Photonics.

[26] Pickworth LA (2016). The National Ignition Facility modular Kirkpatrick-Baez microscope. Rev. Sci. Instrum..

[27] Valdivia MP (2016). An x-ray backlit Talbot-Lau deflectometer for high-energy-density electron density diagnostics. Rev. Sci. Instrum..

[28] Benattar R (1991). Effect of radiation on the time-resolved rear-side emission of laser-illuminated foils at 0.25 μm: Comparison with simulations. Laser and Particle Beams.

[29] Zastrau U (2018). A sensitive EUV Schwarzschild microscope for plasma studies with sub-micrometer resolution. Rev. Sci. Instrum..

[30] Schropp A (2015). Imaging Shock Waves in Diamond with Both High Temporal and Spatial Resolution at an XFEL. Sci. Rep..

[31] Beckwith MA (2017). Imaging at an x-ray absorption edge using free electron laser pulses for interface dynamics in high energy density systems. Rev. Sci. Instrum..

[32] Bullock AB (2006). X-ray induced pinhole closure in point-projection x-ray radiography. J. Appl. Phys..

[33] Faneov AY (2009). Submicrometer-resolution *in situ* imaging of the focus pattern of a soft x-ray laser by color center formation in LiF crystal. Optics Letters.

[34] Baldacchini G (2005). Submicron soft X-ray imaging detectors based on LiF crystals and films: characterization and applications. Rev. Sci. Instrum..

[35] Ustione A (2006). Scanning near-field optical microscopy images of microradiographs stored in Lithium Fluoride films with an optical resolution of λ/12. Appl. Phys. Letters.

[36] Grum-Grzhimailo AN (2017). On the size of the secondary electron cloud in crystals irradiated by hard X-ray photns, Eur. Phys. J. D.

[37] Hampai D (2011). High-resolution X-ray imaging by polycapillary optics and lithium fluoride detectors combination, Eur. Phys. Lett..

[38] Faenov AY (2010). Metrology of Wide Field of View Nano-Thickness Foils’ Homogeneity by Conventional and Phase Contrast Soft X-ray Imaging. Japanese Journal of Applied Physics.

[39] Martinolli E (2006). Fast-electron transport and heating of solid targetsin high-intensity laser interaction measured by K fluorescence. Phys. Rev. E.

[40] Samoylova L (2016). WavePropaGator: interactive framework for X-ray free-electron laser optics design and simulations. J. Appl. Cryst..

[41] Chubar O (2008). Time-dependent FEL wavefront propagation calculations: Fourier optics approach. Nucl. Instr. Meth. Phys. Res. A.

